# Cloning of *Hynobius lichenatus* (Tohoku hynobiid salamander) *p53* and analysis of its expression in response to radiation

**DOI:** 10.1186/s12863-020-00856-0

**Published:** 2020-05-20

**Authors:** Toshiki Kamada, Yumi Une, Kumi Matsui, Shoichi Fuma, Teruo Ikeda, Mariko Okamoto

**Affiliations:** 1 Laboratory of Veterinary Immunology, Department of Veterinary Medicine, School of Veterinary Medicine, Azabu University, 1-17-71 Fuchinobe, Chuo-ku, Sagamihara, Kanagawa 252-5201 Japan; 2 Laboratory of Veterinary Pathology, Faculty of Veterinary Medicine, Imabari campus, Okayama University of Science, 1-3 Ikoinooka, Imabari, Ehime 794-8555 Japan; 3 Laboratory of Veterinary Physiology 1, Department of Veterinary Medicine, School of Veterinary Medicine, Azabu University, 1-17-71 Fuchinobe, Chuo-ku, Sagamihara, Kanagawa 252-5201 Japan; 4 Department of Radioecology and Fukushima Project, Center for Advanced Radiation Emergency Medicine, Quantum Medical Science Directorate, National Institutes for Quantum and Radiological Science and Technology, 4-9-1 Anagawa, Inage-ku, Chiba, 263-8555 Japan

**Keywords:** β-Actin, *GAPDH*, *Hynobius lichenatus* (Japanese Tohoku hynobiid salamander), *p53*, Radiation

## Abstract

**Background:**

Caudata species such as salamanders are easily affected by environmental changes, which can drastically reduce their population. The effects of acute X-rays and chronic γ-irradiation on *Hynobius lichenatus*, the Japanese Tohoku hynobiid salamander, are known. However, the expression of radiation-inducible genes, such as the DNA-damage checkpoint response gene *p53*, has not been analyzed in *H. lichenatus*. This has not occurred because there is no established method for mRNA quantification in *H. lichenatus* due to a lack of information on available nucleotide sequences corresponding to both radiation-inducible genes and endogenous control genes such as *ACTB* (β-actin).

**Results:**

In this study, we aimed to evaluate the effects of radiation on gene expression in *H. lichenatus*. Using RNA extracted from irradiated salamanders, we performed rapid amplification of cDNA ends (RACE) and cloned *H. lichenatus* β-actin, glyceraldehyde-3-phosphate dehydrogenase (*GAPDH*) and *p53*. We confirmed that the cloned cDNAs were able to synthesize salamander proteins by western blotting after transfection into cultured HEK293 cells. Proliferation assays using HEK293 cells stably expressing *H. lichenatus* p53 protein showed that this protein has antiproliferative effects, similar to that of mammalian p53. Furthermore, RT-qPCR analysis using gene-specific primers revealed that *p53* mRNA expression in *H. lichenatus* was upregulated upon exposure to radiation.

**Conclusion:**

Our results suggest that *H. lichenatus* p53 protein take an important role in regulating the cellular responses to various stimuli as mammalian p53 does. Furthermore, our study provides novel data to select appropriate primers to analyze internal control mRNA expression in *H. lichenatus* and to evaluate *p53* expression as a marker of radiation and environmental stimuli.

## Background

Amphibians require both water and land for survival; therefore, they are easily affected by environmental changes, especially those caused by ionizing radiation [1, 2]. Ionizing radiation can generate intracellular free radicals that cause cell damage and decrease DNA integrity in animals. Among amphibians, Caudata species are more sensitive to acute irradiation than Anura species [3, 4]. However, data regarding the effects of exposure to ionizing radiation, especially to chronic low-level radiation, on the survival and reproduction of Caudata species are limited. Further, unlike in mammal species such as humans, molecular analyses, such as gene expression analysis, in Caudata after exposure to ionizing radiation are rarely conducted.

Caudata species such as salamanders are well adapted to cool, damp environments; as these species are highly dependent on water, they prefer to inhabit places with easy access to water. However, Caudata species have poor mobility, often living in the same environment throughout their life. Thus, even minute changes in their environment drastically reduce their population. Therefore, it is assumed that salamanders are readily susceptible to and can easily detect changes in their environment. Moreover, several small salamanders are feared to be in danger of extinction; thus, studies have focused on their ecological behavior and conservation.

Owing to their sensitivity to acute radiation, salamanders can be used as experimental models to study the effects of irradiation. *Hynobius lichenatus*, the Japanese Tohoku hynobiid salamander, mainly lives in the wild and is currently not designated as endangered. *Hynobius lichenatus*, which is endemic to Japan, inhabits the northeast regions of the Honshu Island, such as the Fukushima Prefecture. They live in moist places from low elevations to mountainous regions. Recently, an investigation was conducted on the LD_50_ of acute X-ray radiation on Anura and Caudata species [4] and the effects of the chronic γ- irradiation on *H. lichenatus* by histological examination [5]. Furthermore, dose rate estimations based on activity concentrations of ^134^Cs and ^137^Cs were carried out on *H. lichenatus* specimens that were collected in the Fukushima Prefecture from 2011 to 2013 [6]; however, changes in gene expression were not examined. Radiation is known to induce cell death in mammals, and changes in the expression of genes related to cell death have been reported [7, 8]. Therefore, radiation-induced changes in the expression of cell death-related genes in *H. lichenatus*, a species that is highly sensitive to irradiation, need to be studied.

The tumor suppressor gene, *p53*, is activated in response to various genotoxic stimuli. Under the unstressed condition, cells usually contain very low or undetectable levels of p53 protein due to proteasomal degradation. DNA damage by various stress stimuli results in an increase in p53 stability. Activated p53 induces cell death through apoptosis by halting cell proliferation and the cell cycle [9, 10]. Furthermore, the expression of this gene has been reported to change upon various genotoxic stimuli [11–16], and a similar effect may occur in *H. lichenatus*.

Reverse transcription quantitative polymerase chain reaction (RT-qPCR) is a technique used to examine minute changes in gene expression. However, the *p53* gene sequence in *H. lichenatus* has not been reported and hence, there is no established sequence for mRNA detection. The sequences of endogenous controls in *H. lichenatus*, such as *ACTB* (β-actin) and glyceraldehyde-3-phosphate dehydrogenase (*GAPDH*) gene, which are used to normalize the mRNA expression of a gene of interest in RT-qPCR, are also unknown. Therefore, identifying the sequences of these genes in *H. lichenatus*, particularly their coding regions, is of importance.

In this study, we aimed to (1) investigate the effects of ionizing radiations (X-rays, and γ-rays), and non-ionizing radiation (ultraviolet (UV) light) on *H. lichenatus*, focusing on changes in gene expression; (2) clone and identify the coding sequences of β-actin and *GAPDH* from *H. lichenatus*, which can be used as endogenous controls, and that of *p53*, which can be used as a marker of environmental change; and (3) confirm that these cloned open reading frames can be translated to proteins in transfected cells.

## Results

### Identification of the coding sequences of *H. lichenatus* β-actin, *GAPDH*, and *p53*

**Fig. 1:**
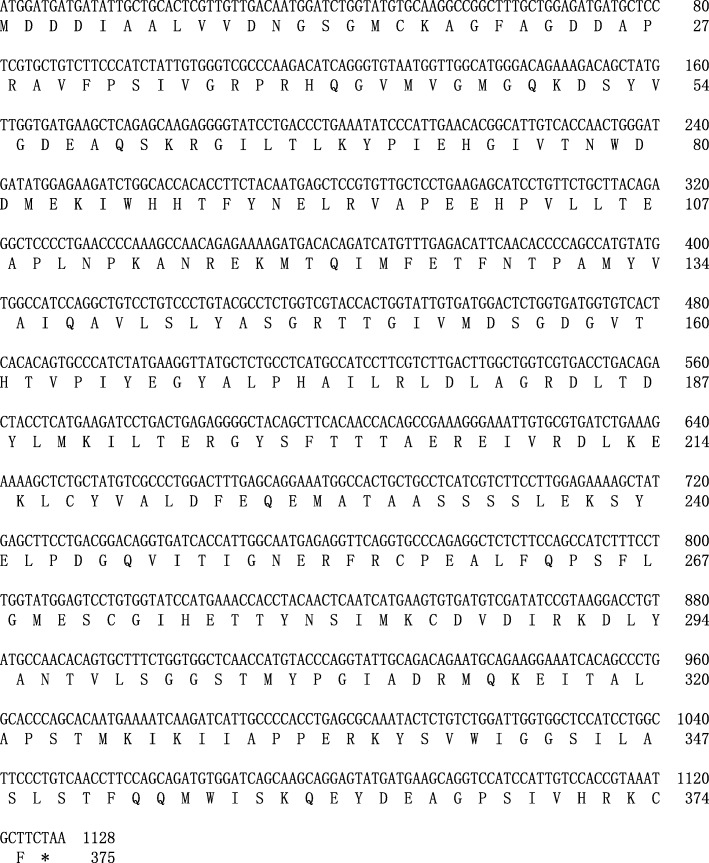
Coding sequence and deduced amino acid sequence of *Hynobius lichenatus* β-actin. The first M and asterisk represent the start codon and termination codon, respectively

**Fig. 2:**
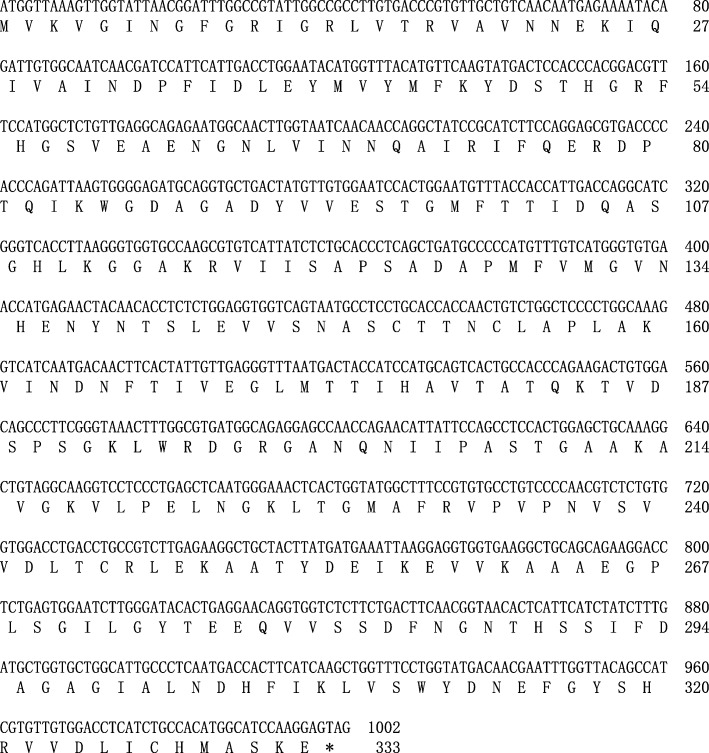
Coding sequence and deduced amino acid sequence of *Hynobius lichenatus GAPDH*. The first M and asterisk represent the start codon and termination codon, respectively

In this study, we cloned the coding sequence of hyβ-actin and salamander *GAPDH* (hyGAPDH) by PCR and RACE. As shown in Fig. 1, the final hyβ-actin cDNA obtained was a 1128-bp open reading frame that encodes a protein of 375 amino acids. The final hyGAPDH cDNA obtained was a 1002-bp open reading frame that encodes a protein of 333 amino acids (Fig. 2). Sequence homology analysis was used to compare the DNA sequence and deduced amino acid sequence of this β-actin or *GAPDH* gene with that of other vertebrates which complete coding sequences have been identified (Tables 1 and 2). The hyβ-actin polypeptide shows extremely high sequence homology (> 97%), and the hyGAPDH peptide has relatively high sequence homology (> 82%), with that of other vertebrates.

**Table 1:** Nucleic acid and amino acid homologies between the β-actin coding region of *Hynobius lichenatus* and that of different vertebrate species. Sequence homology analysis was conducted using GENETYX software (Genetyx, Tokyo, Japan)

β-actin	*Homo sapiens*	*Mus musculus*	*Canis lupus*	*Andrias davidianus*	*Cynops ensicauda*	*Cynops pyrrhogaster*
Nucleic acid (%)	84.8	85.5	84.6	93.9	91.1	88.6
Amino acid (%)	98.9	98.9	98.9	98.9	98.9	97.1

**Table 2:** Nucleic acid and amino acid homologies between the *GAPDH* coding region of *Hynobius lichenatus* and that of different vertebrate species. Sequence homology analysis was conducted using GENETYX software

GAPDH	*Homo sapiens*	*Mus musculus*	*Canis lupus*	*Xenopus laevis*	*Xenopus tropicalis*	*Danio rerio*
Nucleic acid (%)	76.7	78.0	78.8	77.5	79.0	76.3
Amino acid (%)	82.1	81.7	82.9	82.6	81.9	81.7

**Fig. 3:**
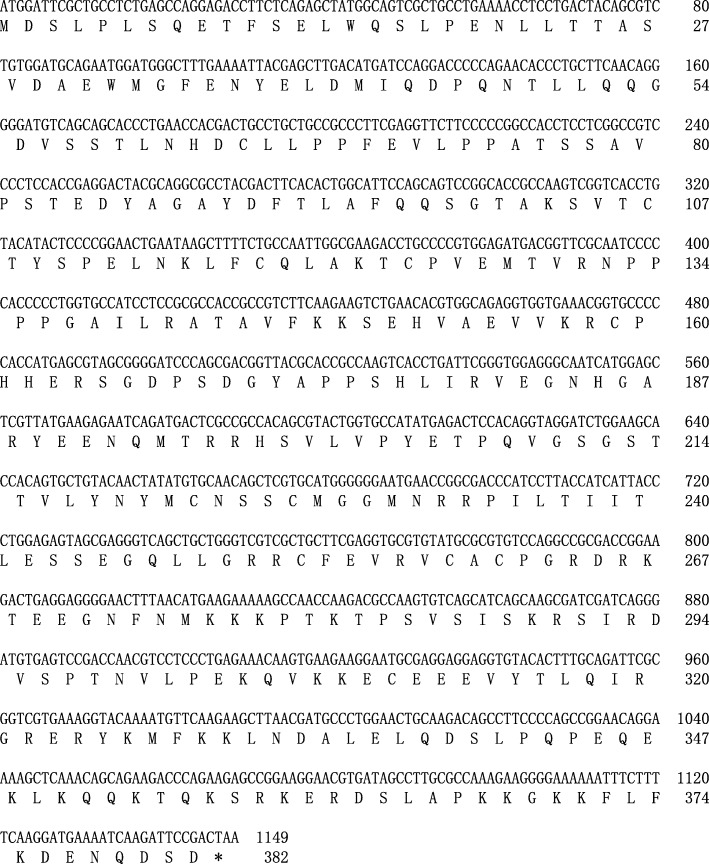
Coding sequence and deduced amino acid sequence of *Hynobius lichenatus p53*. The first M and asterisk represent the start codon and termination codon, respectively

We then cloned the coding sequence of hyp53 by PCR, RACE, and nested PCR. As shown in Fig. 3, the final hyp53 cDNA obtained was a 1149-bp open reading frame encoding a protein of 382 amino acids. Sequence homology analysis was used to compare the DNA sequence and deduced amino acid sequence of this *p53* gene with that of other vertebrates which complete coding sequences have been identified (Table 3). The hyp53 polypeptide has relatively high sequence homology (> 71%) with that of related salamander or newt, but has lower sequence homology (< 52%) with that of mammals.

**Table 3:** Nucleic acid and amino acid homologies between the *p53* coding region of *Hynobius lichenatus* and that of different vertebrate species. Sequence homology analysis was conducted using GENETYX software

p53	*Homo sapiens*	*Mus musculus*	*Canis lupus*	*Xenopus tropicalis*	*Ambystoma mexicanum*	*Cynops orientalis*
Nucleic acid (%)	60.3	59.8	62.2	62.9	70.7	70.8
Amino acid (%)	49.5	49.5	52.0	59.9	71.1	71.4

**Fig. 4:**
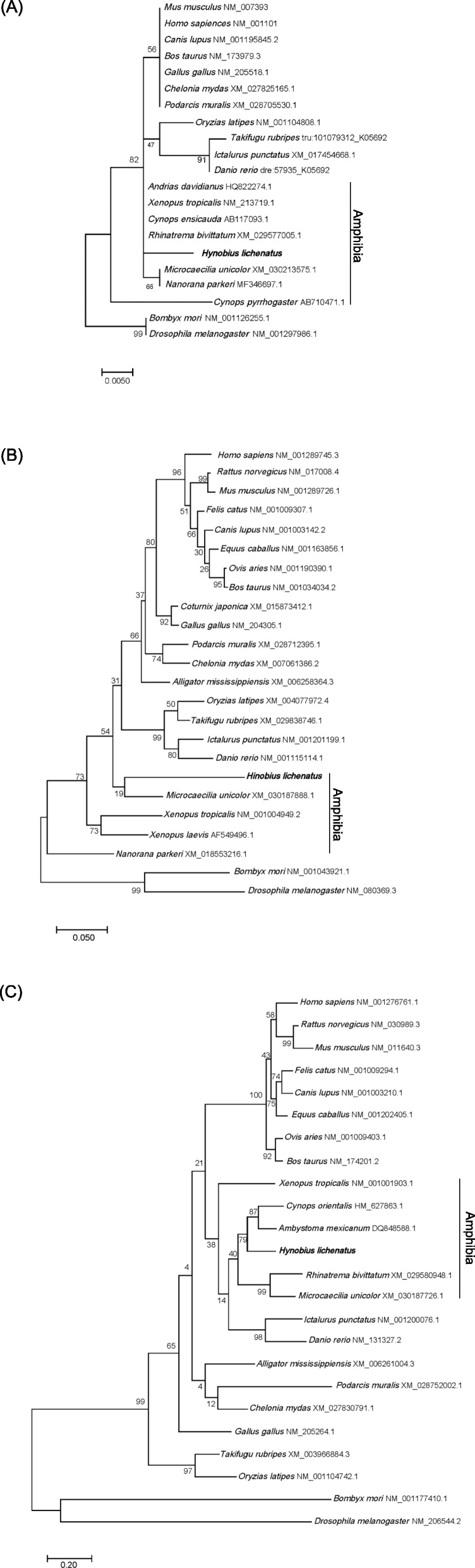
Phylogenetic analysis of β-actin, GAPDH, and p53. Using predicted amino acid sequences, a phylogenetic tree (A, β-actin; B, GAPDH; C, p53) was constructed by the MEGA 10 software using maximum likelihood method. The numbers in this tree indicate the bootstrap value (%) after 1000 replicants

In order to estimate the phylogenetic relationship between hyβ-actin, hyGAPDH, and hyp53 in different species, a phylogenetic tree was generated based on the deduced amino acid sequences of β-actin, *GAPDH*, and *p53* from various mammalia, aves, reptilia, amphibin, teleost fish, and invertebrates (Fig. 4). The amphibian p53 sequences formed a clade separated from those of mammalian and reptile, hyp53 were closest to the *p53* of *Ambystoma mexicanum* and *Cynops orientalis*.

### Expression of *H. lichenatus* proteins in mammalian cells

**Fig. 5:**
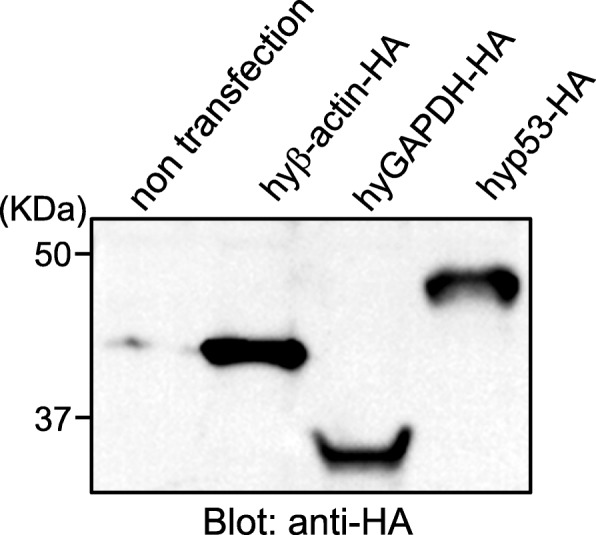
Western blot analysis of *Hynobius lichenatus* proteins in mammalian cells. The expression plasmids (phyβ-actin-HA, phyGAPDH-HA, and phyp53-HA) were transfected into HEK293 cells. Transgene expression was analyzed by western blotting two days after transfection using antibodies against the HA-tag. The original western blot image is shown in Additional file 5A

We next constructed hyβ-actin-, hyGAPDH-, and hyp53-expression plasmids and transfected these plasmids into HEK293 cells to examine whether the corresponding proteins could be synthesized in mammalian cells. The transient expression of exogenous HA-tagged hyβ-actin, hyGAPDH, or hyp53 protein (the HA was fused at the C-terminus of each salamander protein) in HEK293 cells was confirmed by western blotting using an antibody against the HA-tag (Fig. 5). As calculated using ExPASy software (https://web.expasy.org/compute_pi/), the theoretical isoelectric point and molecular weight of hyβ-actin, hyGAPDH, and hyp53 are 5.29 and 41.7 kDa, 5.73 and 36.0 kDa, and 6.68 and 43.1 kDa, respectively. As shown in Fig. 5, western blotting analysis revealed that the hyβ-actin-HA and hyGAPDH-HA protein were detected as a single band at a size consistent with the predicted molecular weight. While the predicted size of hyp53 was 43.1 kDa, we detected the hyp53-HA protein as a single band at an apparent molecular weight of ~ 50 kDa by western blotting. It seems to have undergone modifications, such as glycosylation or ubiquitination, which cause gel mobility shifts. Taken together, these results indicate that the corresponding salamander proteins could be synthesized in mammalian cells from the hyβ-actin, hyGAPDH, and hyp53 cDNA that we cloned. Further, we confirmed that the hyp53-HA protein expressed in HEK293 cells reacts with an antibody against human p53 by immunoprecipitation and western blot analysis (Additional file 2). However, antibodies against human β-actin and GAPDH did not bind to the hyβ-actin-HA and hyGAPDH-HA protein, respectively (data not shown).

### Overexpression of hyp53-HA protein exhibits inhibitory effects on mammalian cell growth

**Fig. 6:**
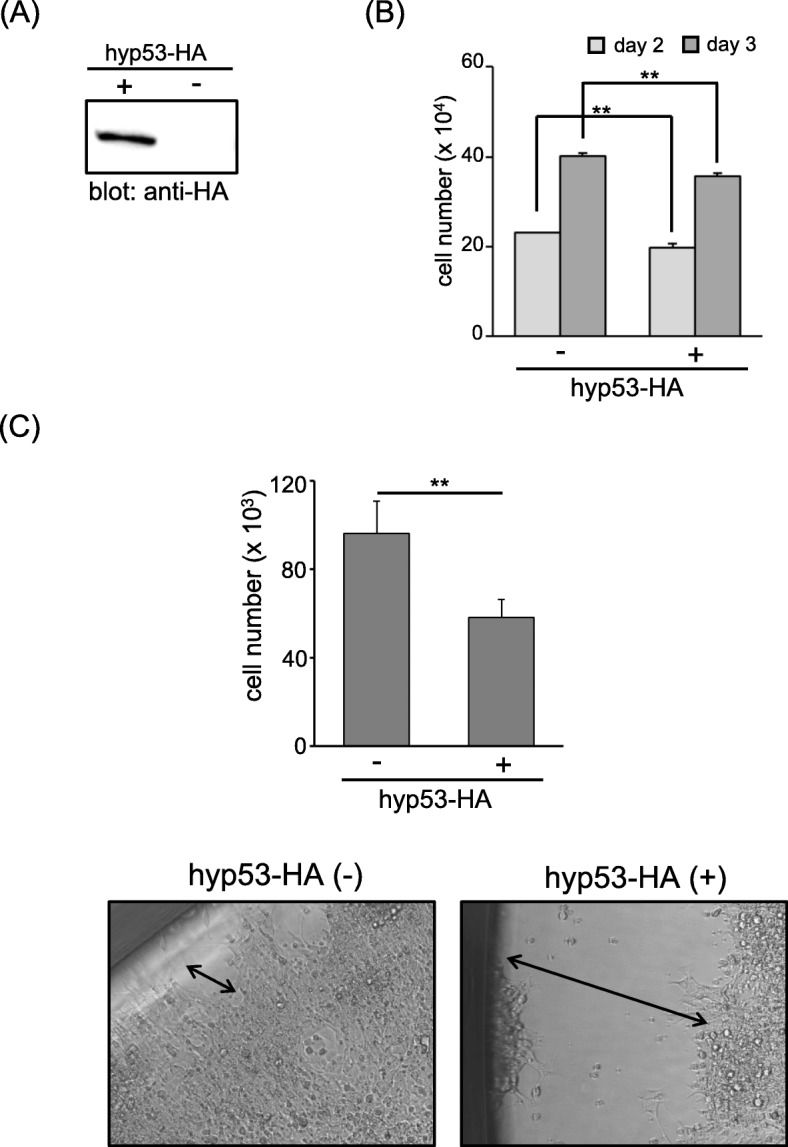
*Hynobius lichenatus* p53 protein exhibits inhibitory effects on mammalian cell proliferation. **a** hyp53-HA protein expression in hyp53-HA/HEK293 stable cell line. The original western blot image is shown in Additional file 5B. **b** Number of viable hyp53-HA/HEK 293 or GFP-HA/HEK293 stable cells after 2–3 days of incubation following serum starvation for 24 h. The data are expressed as mean ± SD (*n* = 4). **P < 0.01. The data shown are representative of three independent experiments. **c** Top: Number of viable hyp53-HA/HEK 293 or GFP-HA/HEK293 stable cells after 7 days of incubation. The data are expressed as mean ± SD (*n* = 4). **P < 0.01. Bottom: Phase-contrast images of the hyp53-HA/HEK 293 and GFP-HA/HEK293 stable cell lines after 7 days of incubation. The double-headed arrow shows the distance from the proliferating cell front to the side of the plate well. The data shown are representative of three independent experiments

Overexpression of wild-type human p53 protein in several human tumor cell lines leads to cell growth suppression [17–20]. To determine whether *H. lichenatus* p53 can also suppress cell proliferation, we introduced hyp53-HA into HEK293 cells via recombinant lentivirus and established a cell line (hyp53-HA/HEK293) that stably expressed the hyp53-HA protein (Fig. 6a). As shown in Fig. 6b, the number of hyp53-HA/HEK293 cells was significantly lower than the number of GFP-HA/HEK293 cells at both days 2 and 3. The difference in cell number between the two stable lines was more apparent after 7 days of incubation in 96-well plates at a cell density of 1 × 10^5^ cells /well (Fig. 6c). Phase-contrast images of the two stable cell lines on day 7 also show that hyp53-HA/HEK293 exhibited less cell proliferation compared with that of GFP-HA/HEK293, as the latter cells had almost reached confluence (Fig. 6c bottom). These results suggest that *H. lichenatus* p53 has the ability to suppress cell proliferation, like mammalian p53.

### Acute radiation increases hyp53 expression in the skin of *H. lichenatus*

**Fig. 7:**
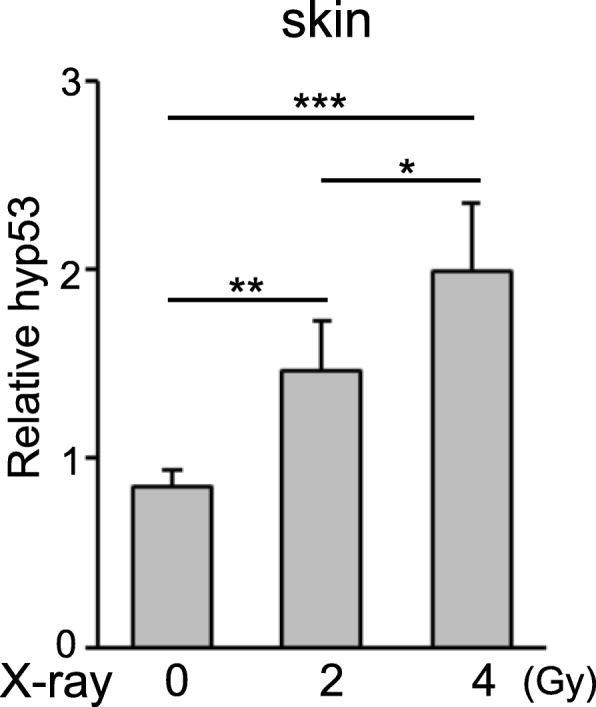
*Hyp53* expression in the skin of *Hynobius lichenatus* is upregulated by acute X-ray radiation. Juveniles of *H. lichenatus* were acutely X-ray irradiated at 0, 2, or 4 Gy and the expression of hyp53 in the skin was analyzed by RT-qPCR. The data are expressed as mean ± SD (*n* = 5). * *P* < 0.05, ** *P* < 0.01, *** *P* < 0.001

The cloning and identification of the coding sequence of *H. lichenatus* β-actin, *GAPDH*, and *p53* enabled us to design primer sets for qPCR to analyze the mRNA expression levels of genes in tissues or cells from *H. lichenatus*. Thus, we examined whether acute X-ray irradiation affects the expression of the *hyp53* gene. Juveniles of *H. lichenatus* were exposed to acute X-ray irradiation at 2 or 4 Gy, and the expression of hyp53 was then analyzed by RT-qPCR. A significant increase in the expression of hyp53 mRNA was observed in the skin of the salamanders after acute X-ray irradiation (Fig. 7). X-ray irradiation with 4 Gy induced significantly higher levels of hyp53 than X-ray irradiation with 2 Gy (Fig. 7). The expression of hyp53 in the spleens of juveniles was also significantly increased after acute X-ray irradiation at a dose of 4 Gy (Additional file 3). The expression of hyp53 was induced in the liver of the salamanders after acute X-ray irradiation, but the difference between non-irradiated and irradiated treatments was not statistically significant (Additional file 3). In addition, hyp53 expression in the skin, spleen, and liver was significantly increased by acute UV radiation (Additional file 4).

### Chronic γ-irradiation increases hyp53 expression in the skin of *H. lichenatus*

**Fig. 8:**
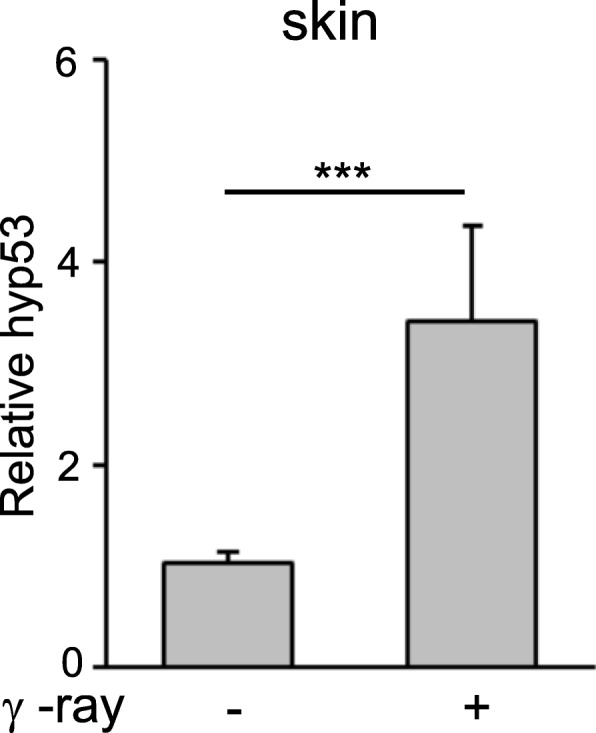
*Hyp53* expression in the skin of *Hynobius lichenatus* is upregulated by chronic γ-irradiation The salamanders were chronically γ-irradiated at with (+) or without (−) 470 μGy h^− 1^ for 740 days and the expression of hyp53 in the skin was analyzed by RT-qPCR. The data are expressed as mean ± SD (*n* = 5). *** *P* < 0.001.

We then examined whether chronic γ-ray irradiation affects hyp53 gene expression. The salamanders were exposed to chronic γ-rays at 470 μGy h^− 1^ for 740 days, and then the expression of hyp53 mRNA was analyzed by RT-qPCR. As shown in Fig. 8, a significant increase in the expression of hyp53 was observed in the skin of the salamanders after this chronic γ-ray irradiation.

## Discussion

We cloned and identified the coding sequence for *H. lichenatus* β-actin, *GAPDH*, and *p53*. We confirmed the expression of salamander proteins in mammalian cells transfected with expression plasmids containing hyβ-actin, hyGAPDH, or hyp53 cDNA that we had cloned. Further, we showed that hyp53 protein stably expressed in mammalian cells inhibits cell proliferation, like mammalian p53. Given this information on the coding sequences of these genes, widespread gene expression analysis of *H. lichenatus* with normalization by internal control is expected to be performed in the future.

Sequence homology analysis of the hyβ-actin gene revealed high DNA and deduced amino acid homology to that of other vertebrates, including higher animals. Furthermore, the hyGAPDH polypeptide shares high sequence homology with the GAPDH from other vertebrates. The results suggest that these genes are conserved across species because they are indispensable for cellular activity and cell structure. On the contrary, a comparison of the DNA and deduced amino acid sequence of *p53* between *H. lichenatus* and mammals showed relatively low sequence homology compared with that of β-actin and *GAPDH*. However, several amino acid residues, which are targets of post-transcriptional modifications and are essential for p53 activation, are conserved in hyp53. This suggests that hyp53 exhibited antiproliferative effects in the hyp53-HA/HEK293 stable cell line by molecular mechanisms similar to those in mammals.

The p53 protein mainly consists of the following three regions: an N-terminal region containing a transactivation domain and a proline-rich domain, a core region containing a DNA-binding domain, and a C-terminal region containing an oligomerization domain and a C-terminal regulatory domain [21, 22]. In the present study, the homology analysis showed that the sequences of these p53 domains were 27% (N-terminal region), 66% (core region), and 36% (C-terminal region) identical between *H. lichenatus* and humans.

Focusing on the N-terminal region of human p53, the phosphorylation of serine residues, Ser15 and Ser20, inhibits the binding of this protein with Mdm2, which ubiquitinates p53 and promotes its degradation [23]. Although the amino acid sequence homology in the N-terminal region is relatively low, these two serine residues are conserved in the sequence of hyp53 (Ser7 and Ser12) and thus are considered important residues regulating p53 function.

With respect to the C-terminal region, multiple C-terminal lysine residues are highly conserved among mammalian p53 proteins. Among them, five lysines located in the C-terminal regulatory domain (Lys370, Lys372, Lys373, Lys381, and Lys382 in human p53) are acetylated by CBP/p300 [23]. Acetylation of those residues prevents the C-terminal regulatory domain from occluding the DNA-binding domain, which promotes an open p53 conformation. This conformational change promotes the binding of p53 to target elements, thus enhancing transcriptional activity and leading to cell cycle arrest [24, 25]. Hyp53 has five lysine residues in its C-terminal regulatory domain (Lys359, Lys367, Lys368, Lys370, and Lys371); thus, it appears that hyp53 also upregulates its transcriptional activity after acetylation of lysine residues in the C-terminus.

Two other lysines located in the oligomerization domain (Lys320 and Lys321 in human p53), have been reported to be a target of FBXO11-mediated neddylation, and the neddylation of p53 leads to inhibition of p53-mediated transcriptional activation [26]. Although the role of p53 neddylation is poorly defined, these two lysine residues are conserved in the sequence of hyp53 (Lys307 and Lys308).

While acetylation of lysine residues in the C-terminus of p53 is important as described above, Tip60- or MOF-mediated acetylation of Lys120 located in the DNA-binding domain plays a crucial role in inducing p53-mediated apoptosis by transcriptionally activating proapoptotic target genes [25, 27, 28]. Lys164 in human p53, another lysine residue within the DNA-binding domain, is a target of acetylation by CBP/p300 [21]. Studies using knockin mice with mutations at Lys117, Lys161, and Lys162, analogous to double mutations at Lys120 and Lys164 in human p53, have shown that loss of acetylation at these lysine residues abolishes p53-mediated cell cycle arrest, senescence, and apoptosis [21, 29]. Both Lys120 and Lys164 are well conserved among species, and are also conserved in the sequence of hyp53 (Lys103 and Lys147).

Another post-translational modification of p53 is poly-ADP-ribosylation. In human p53, two glutamate residues (Glu258 and Glu271) within the DNA-binding domain are the poly-ADP-ribosylation target of PARP1 [30]. PARP1-mediated poly-ADP-ribosylation of these amino acids has been shown to block the interaction between p53 and the nuclear export receptor Crm1, leading to nuclear accumulation of p53 [30]. These two glutamate residues are highly conserved among species, and are also conserved in the sequence of hyp53 (Glu242 and Glu255).

We showed in this study that hyp53 mRNA levels increase upon exposure to radiation. The induction of p53 at the gene level has been reported upon various genotoxic stimuli including UV [15] and ionizing radiation [16] and our present data indicate that the same effect occurs in *H. lichenatus*. We did not analyze radiation-induced mRNA expression of other mediators that are located upstream or downstream of hyp53. Thus, further studies are required to examine whether activated hyp53 induces target genes after radiation exposure via a pathway similar to that in mammals.

It is well known that post-transcriptional modifications of p53 caused by DNA damage lead to a rise of p53 protein levels in mammals. Furthermore, in zebrafish, mRNA and protein levels of p53 increase upon exposure to radiation [31], and p53 protein enhances the translation of its own mRNA in response to UV irradiation [32]. We did not investigate hyp53 expression changes at the protein level. However, we found that hyp53-HA exogenously overexpressed in mammalian cells could be detected using an antibody against human p53; further studies are needed to examine whether endogenous hyp53 (basal and radiation-induced) can also be detected using this antibody.

In regard to UVC radiation, *p53* expression is increased in cultured cells upon exposure to UVC [33–36]. On the other hand, data regarding the change of *p53* expression upon UVC exposure in vivo are limited. Campbell et al reported a limited expression of p53 protein in the upper layers of the epidermis in biopsies of UVC-irradiated skin, with diffuse p53 expression throughout the epidermis by UVB radiation [37]. The difference between the expression patterns observed with UVC and UVB was suggested to be due to the differences in UV penetration into skin. In the present study, UVC radiation increased hyp53 mRNA levels in the spleen and liver. We did not examine whether UVC has a gradient effect with increasing depth in these tissues; therefore, the possibility that there are differences in *p53* mRNA expression at the surface and in deep parts of these tissues cannot be excluded.

In a previous study, the salamanders were chronically exposed to γ-irradiation from the embryonic to juvenile stages. At higher dose rates (4600 or 18,000 μGy h^− 1^), severe effects, including growth inhibition, delayed metamorphosis, morphological aberrations, subcutaneous hemorrhage, and organ damage, were observed, and all irradiated salamanders died [5]. In contrast, at 490 μGy h^− 1^ or lower dose rates, there were no significant changes in the survival, weight, and average age at metamorphosis of the juveniles compared with those of the non-irradiated controls. Further, no histological changes were observed in the liver, spleen, intestine, and skin at lower dose rates [5]. Our results showed that the mRNA expression of hyp53 in the skin was increased in chronically γ-irradiated salamanders at a relatively low dose rate (470 μGy h^− 1^). It is possible that hyp53 is transcriptionally induced after chronic γ-irradiation, and the synthesized hyp53 protein contributes to DNA damage repair, thus preventing tissue damage. After acute X-ray and UV irradiation, hyp53 mRNA was upregulated in at least the skin and spleen without visible tissue damage. However, the possibility that hyp53 is induced before tissue damage or morphological abnormality cannot be excluded and further studies are required in this regard. Nevertheless, based on our results that hyp53 expression is induced upon exposure to radiation, *H. lichenatus* can be assumed to upregulate hyp53 expression in response to other environmental stimuli or changes.

## Conclusions

We cloned and verified the integrity of *H. lichenatus p53* gene, as well as the housekeeping genes, β-actin and *GAPDH*. We showed that hyp53 expression suppressed the proliferation of cultured cells and that hyp53 mRNA responded to radiation, similar to that observed in mammals. The sequences of the salamander genes that we identified and the RT-qPCR method that we established to detect hyp53 mRNA expression in this study will provide a viable approach to survey environmental changes. Furthermore, our study provides data to select appropriate internal control primers for mRNA expression analysis in *H. lichenatus*. This will enable us to evaluate the mRNA expression of genes of interest other than hyp53, contributing to the progress of molecular analysis of *H. lichenatus*.

## Methods

### Animals

Sampling of wild *H. lichenatus* was carried out in a ditch in the mountain forest (natural and no-man’s forest, non-specific forest owners) of the Aizu District, Fukushima, Japan. Our previous study confirmed that radiation exposures due to the Fukushima nuclear accident were negligible in this District [6]. Juvenile and adult *H. lichenatus* were maintained in individual aquaria at 15 °C (for juveniles) or 0–22 °C (for adults). The study was approved by the Azabu University Animal Research Ethics Committee (Approval No. 150316–6). All procedures involving animals were performed in compliance with the experimental animal guidelines of Azabu University.

### Irradiation of *H. lichenatus*

The salamanders were exposed to acute X-rays at a dose of 2 or 4 Gy using an X-ray generator (TITAN-320, Shimadzu Corp., Japan) and maintained for 2 weeks before tissue sampling. Τhe salamanders were exposed to chronic γ-radiation at a dose rate of 470 μGy h^− 1^ for 740 days using a ^137^Cs source. After γ-irradiation, the salamanders were maintained for 3 days before tissue sampling.

### First-strand cDNA synthesis

The irradiated or control (non-irradiated) salamanders were euthanized with 10% eugenol (Fujifilm-Wako, Osaka, Japan), and the skin, spleen, and liver were isolated. The tissue samples were washed with Hank’s balanced salt solution (Sigma-Aldrich, St. Louis, MO, USA) and homogenized manually using small pestles. The homogenized tissues were then suspended in RiboZol™ RNA Extraction Reagent (AMRESCO, Solon, OH, USA) for total RNA extraction, according to the manufacturer’s instructions. First-strand cDNA was synthesized from the total RNA using oligo (dT) primer and SuperScript III Reverse Transcriptase (Thermo Fisher Scientific, Waltham, MA, USA).

### Double-strand cDNA synthesis and adaptor ligation

Double-strand cDNA for rapid amplification of cDNA ends (RACE) was synthesized from the total RNA extracted from skin using the PrimeScript Double Strand cDNA Synthesis Kit (Takara, Kusatsu, Japan). The RACE adaptor was synthesized by annealing two oligonucleotides (5′ CTAATACGACTCACTATAGGGCTCGAGCGGCCGCCCGGGCAGGT 3′ and 5′ p- CCATCCTAATACGACTCACTATAGGGC-a 3′, where p is 5′-phosphorylation and a is 3΄-amination) and ligated to the blunt ends of the double-strand cDNA using the Mighty Mix DNA Ligation Kit (Takara).

### Cloning of coding sequences of *H. lichenatus* β-actin, *GAPDH*, and *p53*

A β-actin cDNA fragment (nucleotide positions 749–1086) was amplified from the first-strand cDNA mentioned above using primers (Nos. 1 and 2 in Additional file 6) directed against conserved β-actin sequences among other closely related salamanders. The polymerase chain reaction (PCR) was performed according to the protocol No. 1 in Additional file 7. The PCR products were subcloned into the pCR-Blunt II-TOPO vector (Thermo Fisher Scientific, Waltham, MA, USA) and sequenced by Fasmac Co., Ltd. (Atsugi, Japan). After identifying the nucleotide sequence, reverse (nucleotide positions 777–796, No. 3 in Additional file 6) and forward (nucleotide positions 934–956, No. 4 in Additional file 6) primers for RACE were synthesized accordingly. To obtain the 5′ and 3′ ends of the β-actin cDNA, RACE was performed with adaptor-ligated double-stranded cDNA as the template (protocol No. 2 or 3 in Additional file 7). The PCR products were subcloned and sequenced as mentioned above.

The *GAPDH* coding sequence (nucleotide positions 1–1002) was amplified from the first-strand cDNA mentioned above using degenerate primers (Nos. 6 and 7 in Additional file 6) directed against conserved *GAPDH* sequences among other closely related salamanders. PCR was performed according to the protocol No. 4 in Additional file 7. The PCR products were subcloned and sequenced as mentioned above.

The *p53* cDNA fragment (nucleotide positions 658–824) was amplified from the first-strand cDNA mentioned above using degenerate primers (Nos. 8 and 9 in Additional file 6) directed against conserved *p53* sequences among other closely related salamanders. PCR was performed according to the protocol No. 5 in Additional file 7. The PCR products were subcloned and sequenced as mentioned above. After identifying the nucleotide sequence, reverse primers corresponding to nucleotide positions 797–818 and 792–811 (Nos. 10 and 12 in Additional file 6, respectively) and forward primers corresponding to nucleotide positions 711–734 and 720–740 (Nos. 13 and 14 in Additional file 6, respectively) for RACE were synthesized accordingly. To obtain the 5′ end of the *p53* cDNA, the first RACE PCR was performed with adaptor-ligated double-stranded cDNA as the template according to the protocol No. 6 in Additional file 7. One-tenth volume of the PCR products was then used for nested PCR (protocol No. 7 in Additional file 7). To obtain the 3′ end of the *p53* cDNA, the first RACE PCR was performed according to the protocol No. 8 in Additional file 7, followed by nested PCR (protocol No. 9 in Additional file 7). These PCR products were purified using ExoSAP-IT for PCR Product Clean-Up (Takara) and sequenced.

### Phylogenic analysis

Multiple sequence alignments and construction of phylogenetic tree were performed with the Molecular Evolutionary Genetic Analysis (MEGA) 10 software using maximum likelihood method with 1000 bootstrap replicates.

### Plasmid construction

The full-length coding sequence of the β-actin cDNA (1–1128) was generated by PCR with the first-strand cDNA as the template (protocol No. 10 in Additional file 7). The PCR product was digested using the restriction enzymes HindIII (New England Biolabs, Ipswich, MA, USA) and XhoI (Takara), and then cloned into pcDNA3-HA (a plasmid obtained by inserting the HA-tag sequence into pcDNA3 [Thermo Fisher Scientific]; provided by Dr. Masaru Murakami, Azabu University) to obtain p*Hynobius* (hy) β-actin-HA.

The full-length coding sequence of the *GAPDH* cDNA (1–1002) was generated by PCR with the first-strand cDNA as the template (protocol No. 11 in Additional file 7). The PCR product was digested using the restriction enzymes Hind III and XhoI, and then cloned into pcDNA3-HA to obtain phyGAPDH-HA.

To obtain the full-length coding sequence of the *p53* cDNA (1–1149), the initial PCR was performed according to the protocol No. 12 in Additional file 7. One-tenth volume of the PCR product was then used for nested PCR (protocol No. 13 in Additional file 7). The PCR product was digested using the restriction enzymes KpnI (New England Biolabs) and XhoI (Takara), and then cloned into pcDNA3-HA to obtain phyp53-HA.

The lentivirus vector expressing HA-tagged p53 protein, phyp53-HA-LVSIN, was constructed by inserting the p53-HA cDNA fragment into pLVSIN-CMV-Neo (Takara). The p53-HA fragment was generated by PCR with phyp53-HA as the template (protocol No. 14 in Additional file 7). The PCR-amplified product was digested using EcoRI (New England Biolabs) and NotI (Takara), and then cloned into pLVSIN-CMV-Neo. The lentivirus vector expressing GFP protein, pGFP-LVSIN, was generated as the control lentivirus vector. The nucleotide sequences of all PCR-amplified products were verified by Fasmac Co., Ltd.

### RT-qPCR

RT-qPCR was performed on a 7500 Real-Time PCR System (Thermo Fisher Scientific) using the DyNAmo ColorFlash SYBR Green qPCR Kit (Thermo Fisher Scientific) according to the protocol No. 15 or 16 in Additional file 7. The mRNA expression of hyp53 was calculated using hyβ-actin as the normalization control.

### Cell culture

HEK293 (human embryonic kidney) cells were obtained from the Human Science Research Resources Bank (Ibaraki, Japan) and cultured as reported previously [38]. The Lenti-X 293 T cell line was purchased from Takara. This cell line was cultured in Dulbecco’s modified Eagle’s medium (DMEM) (Fujifilm-Wako) containing 10% heat-inactivated Tet system approved fetal bovine serum (Takara) and 100 units/mL penicillin/streptomycin (Fujifilm-Wako), and maintained in 5% CO_2_ atmosphere at 37 °C.

### Transfection and western blotting

HEK293 cells (5 × 10^5^ cells/well) were plated in six-well plates the day before transfection. DNA transfection was performed using X-treme GENE™ HP DNA Transfection Reagent (Sigma-Aldrich) with 2 μg of expression plasmid (phyβ-actin-HA, phyGAPDH-HA, or phyp53-HA). After 2 days of incubation at 37 °C, the cells were washed with PBS and lysed in RIPA Buffer (Fujifilm-Wako) containing Halt™ Protease and Phosphatase Inhibitor Cocktail (Thermo Fisher Scientific). Western blotting was performed as reported previously [39]. In brief, one tenth volume of the cell lysate was resolved by sodium dodecyl sulfate polyacrylamide gel electrophoresis (SDS-PAGE) and transferred onto an Immobilon-P polyvinylidene difluoride (PVDF) membrane (Merck-Millipore Darmstadt, Germany). The blotted membrane was probed with an antibody against the HA-tag (H3663, 1: 2000; Sigma-Aldrich) followed by incubation with horseradish peroxidase-conjugated secondary antibody (7072–1; 1:3000; Cell Signaling Technology, Danvers, MA, USA). The labeled proteins were visualized using Chemi-Lumi One substrate (Nacalai Tesque, Kyoto, Japan) and analyzed using the ImageQuant™ LAS4000 imaging system (GE Healthcare, Little Chalfont, United Kingdom).

### Generation of stably transfected cell lines

Lenti-X 293 T cells (2 × 10^6^ cells/dish) were plated in a BioCoat Collagen I Cellware 35 mm dish (BD Biosciences, Franklin Lakes, NJ, USA) the day before transfection. DNA transfection was performed using the Lenti-X HTX Packaging System (Takara) with phyp53-HA-LVSIN or pGFP-LVSIN according to the manufacturer’s instruction. After 2 days of incubation at 37 °C, the lentivirus vector-containing supernatant was harvested. Lenti-X GoStix (Takara) were used to rapidly confirm the presence of lentivirus. The lentivirus containing supernatant was transferred to the BioCoat Fibronectin 35 mm dish (BD Biosciences) in order for the lentivirus vector to bind to the fibronectin coating on the dish. After a 5-h incubation at 37 °C, HEK293 cells (with 8 μg/mL hexadimethrine bromide, Sigma-Aldrich) were plated to the lentivirus vector-bound dish and incubated for 2 days at 37 °C. The vector-transduced cells were selected by culturing the cells in G418 (800 μg/mL, Nacalai Tesque)-containing medium. After 7 days, the cells were harvested and the expression of hyp53-HA protein was confirmed by western blotting as mentioned above.

### Cell proliferation

The hyp53-HA/HEK293 stable cell line or control GFP/HEK293 stable cell line (8 × 10^4^ cells/well) was seeded in Celltight C-1 24-well plates (Sumitomo Bakelite, Tokyo, Japan) and incubated overnight at 37 °C. Serum starvation was performed by changing the culture medium to serum free DMEM. After 24 h of serum starvation, the cells were cultured in DMEM containing 5% fetal bovine serum. After 2–3 days, the cells were harvested and viable cells were counted using 0.5% Trypan Blue Stain Solution (Nacalai Tesque). To examine cell proliferation at high cell density, the hyp53-HA/HEK293 or control GFP/HEK293 stable cell line (1 × 10^5^ cells/well) was seeded in 96-well plates and incubated for 7 days at 37 °C. Cell proliferation was observed under a microscope (EVOS FLoid Cell Imaging Station, Thermo Fisher Scientific) and the cells were counted as mentioned above.

### Statistical analysis

The data were statistically analyzed with a two-tailed Student’s *t* tests or one-way analysis of variance, followed by Dunnett’s multiple comparison tests using GraphPad Prism (GraphPad Software, La Jolla, CA, USA). The data are presented as mean ± standard deviations. Results with a *P* value < 0.05 were considered statistically significant.

## Supplementary information

**Additional file 1.** Additional methods.

**Additional file 2.** Immunoprecipitation and western blot analysis of hyp53-HA transiently expressed in mammalian cells. HEK293 cells were transiently transfected with or without phyp53-HA for 2 days. Cell lysates were immunoprecipitated (IP) with anti-HA and western blotted with anti-human p53 (hp53) antibody. “Input” indicates the input protein lysate.

**Additional file 3 **Hyp53 expression in the spleen and liver of *Hynobius lichenatus* after acute X-ray radiation. *Hynobius lichenatus* were acutely X-ray irradiated at 2 or 4 Gy and the expression of hyp53 in the spleen and liver was analyzed by qPCR. The data are expressed as mean ± SD (*n* = 5). * *P* < 0.05.

**Additional file 4 **Hyp53 expression in *Hynobius lichenatus* is upregulated by UV radiation. *Hynobius lichenatus* (*n* = 1) were acutely UV-C irradiated (8000 J/m^2^) and the expression of hyp53 in the skin, spleen, and liver was analyzed by qPCR. (PPTX 37 kb)

**Additional file 5.** The original images of western blot analysis. (A) The original image of western blot analysis showed in Fig. 5. (B) The original image of western blot analysis showed in Fig. 6a

**Additional file 6.** Primers used in this study.

**Additional file 7.** PCR and RT-qPCR protocols used in this study.

## Data Availability

The datasets used during the current study are available in the NCBI (https://www.ncbi.nlm.nih.gov/). GenBank accession numbers of the β-actin are: human (*Homo sapiens*), NM_001101; mouse (*Mus musculus*), NM_007393; dog (*Canis lupus*), NM_001195845.2; Chinese giant salamander (*Andrias davidianus*), HQ822274.1; sword-tail newt (*Cynops ensicauda*), AB117093.1; Japanese fire belly newt (*Cynops pyrrhogaster*), AB710471.1; cattle (*Bos taurus*), NM_173979.3; chicken (*Gallus gallus*), NM_205518.1; green sea turtle (*Chelonia mydas*), XM_027825165.1; common wall lizard (*Podarcis muralis*), XM_028705530.1; Japanese medaka (*Oryzias latipes*), NM_001104808.1; Japanese pufferfish (*Takifugu rubripes*), XM_003964421.3; channel catfish (*Ictalurus punctatus*), XM_017454668.1; zebrafish (*Danio rerio*), NM_131031.2; western clawed frog (Xenopus tropicalis), NM_213719.1; two-lined caecilian (*Rhinatrema bivittatum*), XM_029577005.1; tiny cayenne caecilian (*Microcaecilia unicolor*), XM_030213575.1; high himalaya frog (*Nanorana parkeri*), XM_018571725.1; domestic silkworm (*Bombyx mori*), NM_001126255.1; fruit fly (*Drosophila melanogaster*), NM_001297986.1. GenBank accession numbers of *GAPDH* are: human (*Homo sapiens*), NM_001289745.3; mouse (*Mus musculus*), NM001289726.1; dog (*Canis lupus*), NM_001003142.2; African clawed frog (*Xenopus laevis*), AF549496.1; western clawed frog (*Xenopus tropicalis*), NM001004949.2; zebrafish (*Danio rerio*), NM001115114.1; rat (*Rattus norvegicus*), NM_017008.4; cat (*Felis catus*), NM_001009307.1; horse (*Equus caballus*), NM_001163856.1; sheep (*Ovis aries*), NM_001190390.1; cattle (*Bos taurus*), NM_001034034.2; Japanese quail (*Coturnix japonica*), XM_015873412.1; chicken (*Gallus gallus*), NM_204305.1; green sea turtle (*Chelonia mydas*), XM_007061386.2; common wall lizard (*Podarcis muralis*), XM_028712395.1; American alligator (*Alligator mississippiensis*), XM_006258364.3; Japanese medaka (*Oryzias latipes*), XM_004077972.4; Japanese pufferfish (*Takifugu rubripes*), XM_029838746.1 channel catfish (*Ictalurus punctatus*), NM_001201199.1; tiny cayenne caecilian (*Microcaecilia unicolor*), XM_030187888.1; high himalaya frog (*Nanorana parkeri*), XM_018553216.1; domestic silkworm (*Bombyx mori*), NM_001043921.1; fruit fly (*Drosophila melanogaster*), NM_080369.3. GenBank accession numbers of *p53* are: human (*Homo sapiens*), NM001276761.1; mouse (*Mus musculus*), NM011640.3; dog (*Canis lupus*), NM001003210.1; western clawed frog (*Xenopus tropicalis*), NM001001903.1; axolotl (*Ambystoma mexicanum*), DQ848588.1; Chinese fire belly newt (*Cynops orientalis*), HM627863.1; rat (*Rattus norvegicus*), NM_030989.3; cat (*Felis catus*), NM_001009294.1; horse (*Equus caballus*), NM_001202405.1; sheep (*Ovis aries*), NM_001009403.1; cattle (*Bos taurus*), NM_174201.2; two-lined caecilian (*Rhinatrema bivittatum*), XM_029580948.1; tiny cayenne caecilian (*Microcaecilia unicolor*), XM_030187726.1; channel catfish (*Ictalurus punctatus*), NM_001200076.1; zebrafish (*Danio rerio*), NM_131327.2; American alligator (*Alligator mississippiensis*), XM_006261004.3; green sea turtle (*Chelonia mydas*), XM_027830791.1; common wall lizard (*Podarcis muralis*), XM_028752002.1; chicken (*Gallus gallus*), NM_205264.1; Japanese medaka (*Oryzias latipes*), NM_001104742.1; Japanese pufferfish (*Takifugu rubripes*), XM_003966884.3; domestic silkworm (*Bombyx mori*), NM_001177410.1; fruit fly (*Drosophila melanogaster*), NM_206544.2.

## Abbreviations

DMEM: Dulbecco’s modified Eagle’s medium
FBS: Fetal bovine serum
GAPDH: Glyceraldehyde-3-phosphate dehydrogenase
GFP: Green fluorescent protein
HA: Hemagglutinin
HEK: Human embryonic kidney
MEGA: Molecular Evolutionary Genetic Analysis
PAGE: Polyacrylamide gel electrophoresis
PBS: Phosphate buffered saline
PVDF: Polyvinylidene difluoride
RACE: Rapid amplification of cDNA ends
RIPA: Radioimmunoprecipitation assay
RT-qPCR: Reverse transcription-quantitative polymerase chain reaction
SDS: Sodium dodecyl sulfate
UV: Ultraviolet
