# Supplementary material for: Cloning of Hynobius lichenatus (Tohoku hynobiid salamander) p53 and analysis of its expression in response to radiation
Source: BMC Genet. 2020 May 20;21:53. doi: 10.1186/s12863-020-00856-0 (PMC7238597; doi:10.1186/s12863-020-00856-0)
Supplement: Supplementary file 1 — Additional file 1. Additional methods. [file 12863_2020_856_MOESM1_ESM.docx]

Additional methods

Immunoprecipitation

The phyp53-HA expression plasmids were transfected into HEK293 cells. The cells were harvested at day 2 after transfection, washed with PBS, and lysed in RIPA Buffer (Fujifilm-Wako) containing the Halt^TM^ Protease and Phosphatase Inhibitor Cocktail (Thermo Fisher Scientific). Immunoprecipitation of the HA-fused protein was performed using the HA-tagged Magnetic PURIFICATION KIT (MBL, Nagoya, Japan) according to the manufacturer’s instruction. The immunoprecipitated proteins were separated by SDS-PAGE. Western blot assays were performed using an antibody against human p53 (sc-6243, 1: 400; Santa Cruz Biotechnology, Dallas, TX, USA) followed by incubation with the horseradish peroxidase-conjugated secondary antibody (7071-1; 1:5,000; Cell Signaling Technology) as described in the Material and Methods.

UV irradiation of *Hynobius lichenatus*

A juvenile of *H. lichenatus* was exposed to UV (8000 J/m^2^) using a XL-1000J UV Crosslinker (Spectronics, NY, USA) and maintained overnight before tissue sampling.
