# Supplementary figures and images for: Cloning of Hynobius lichenatus (Tohoku hynobiid salamander) p53 and analysis of its expression in response to radiation
Source: BMC Genet. 2020 May 20;21:53. doi: 10.1186/s12863-020-00856-0 (PMC7238597; doi:10.1186/s12863-020-00856-0)

## Slide 1
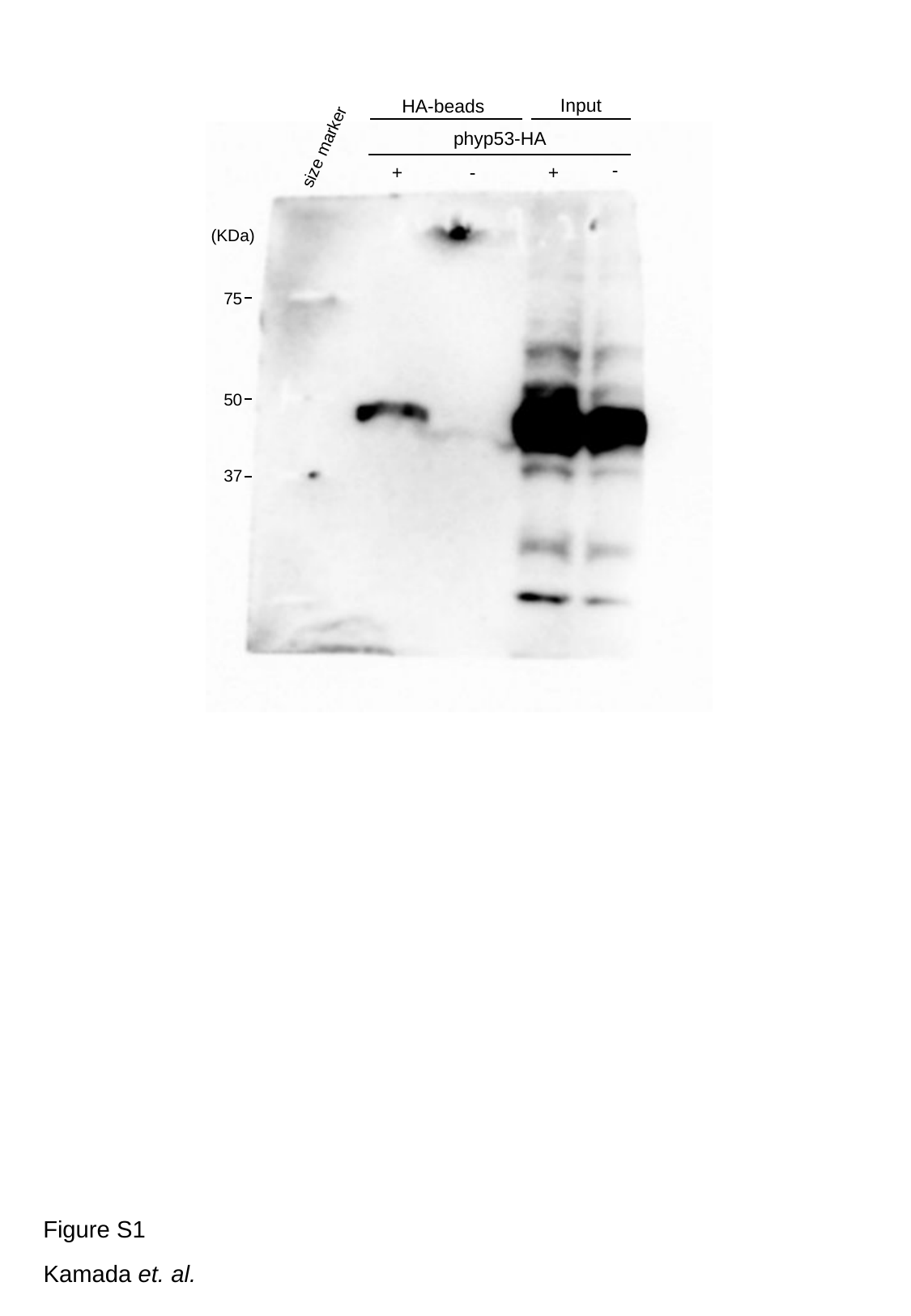

Input
HA-beads
phyp53-HA
size marker
-
+
+
-
(KDa)
75
50
37
Figure S1
Kamada et. al.

Supplement: Supplementary file 2 — Additional file 2. Immunoprecipitation and western blot analysis of hyp53-HA transiently expressed in mammalian cells. HEK293 cells were transiently transfected with or without phyp53-HA for 2 days. Cell lysates were immunoprecipitated (IP) with anti-HA and western blotted with anti-human p53 (hp53) antibody. “Input” indicates the input protein lysate. [file 12863_2020_856_MOESM2_ESM.pptx]

## Slide 1
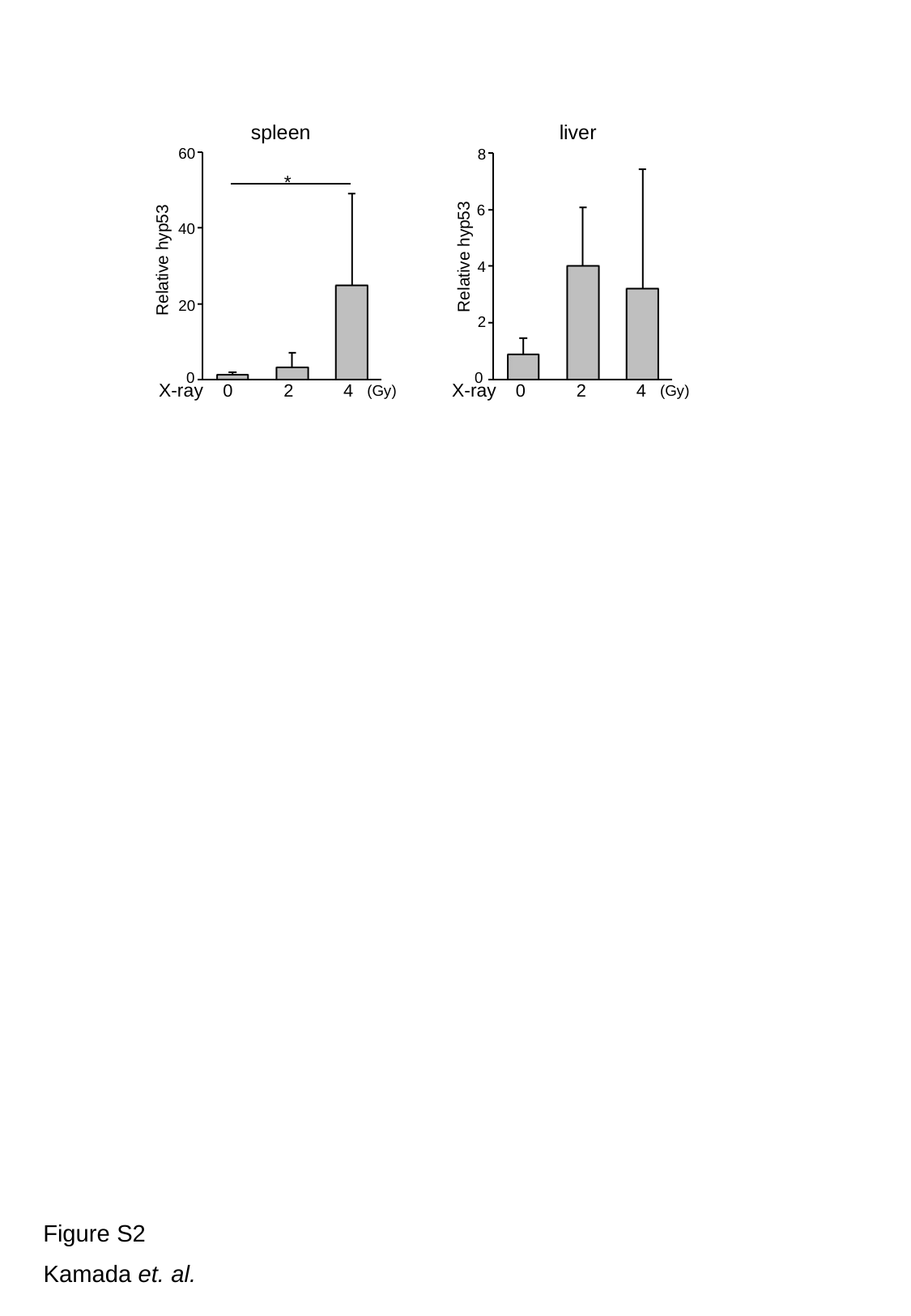

spleen
60
*
40
Relative hyp53
20
0
0
2
4
(Gy)
liver
8
6
Relative hyp53
4
2
0
0
2
4
(Gy)
X-ray
X-ray
Figure S2
Kamada et. al.

Supplement: Supplementary file 3 — Additional file 3 Hyp53 expression in the spleen and liver of Hynobius lichenatus after acute X-ray radiation. Hynobius lichenatus were acutely X-ray irradiated at 2 or 4 Gy and the expression of hyp53 in the spleen and liver was analyzed by qPCR. The data are expressed as mean ± SD (n = 5). * P < 0.05. [file 12863_2020_856_MOESM3_ESM.pptx]

## Slide 1
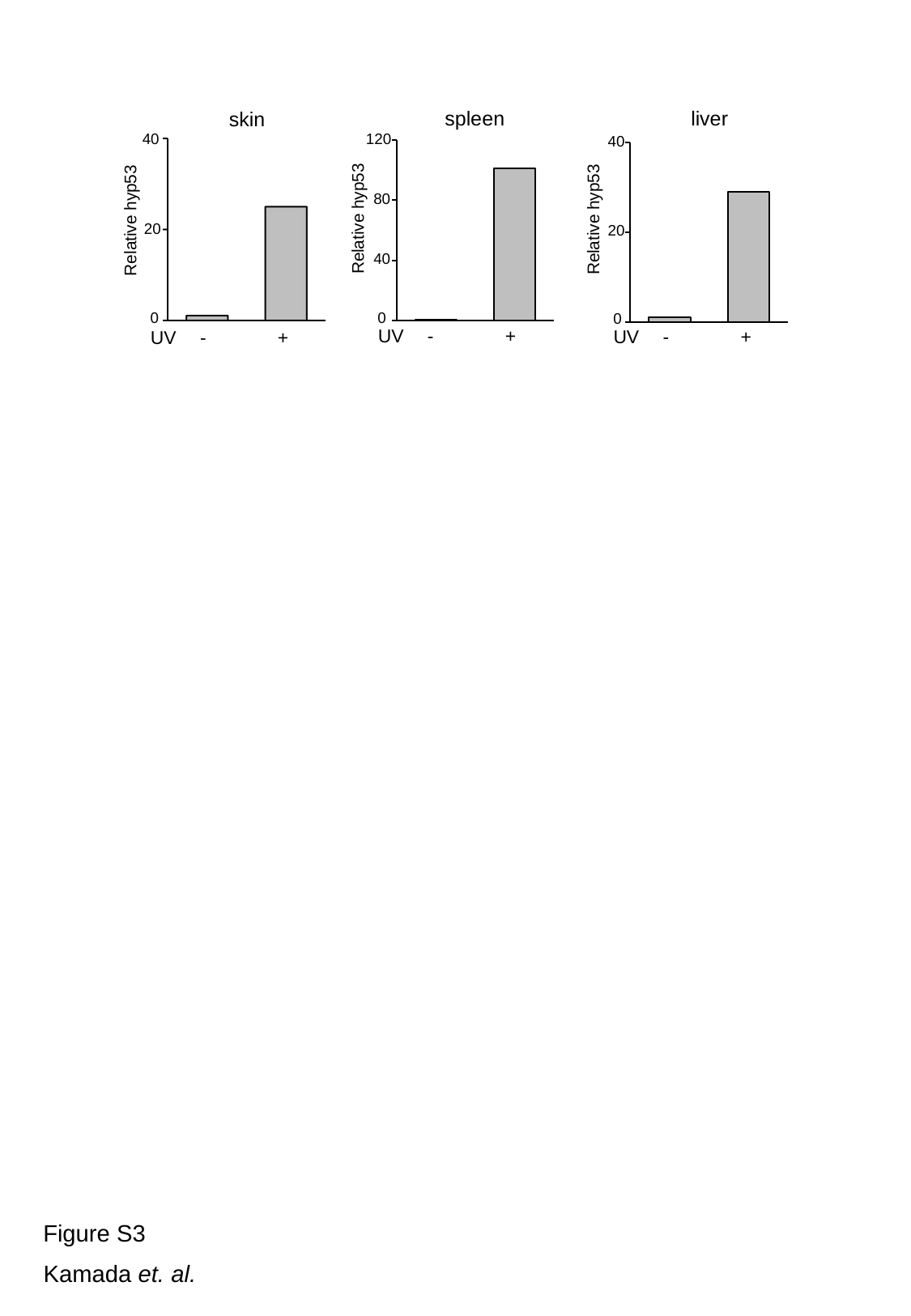

spleen
120
80
Relative hyp53
40
0
-
+
UV
liver
40
20
0
Relative hyp53
-
+
UV
skin
40
Relative hyp53
20
0
-
+
UV
Figure S3
Kamada et. al.

Supplement: Supplementary file 4 — Additional file 4 Hyp53 expression in Hynobius lichenatus is upregulated by UV radiation. Hynobius lichenatus (n = 1) were acutely UV-C irradiated (8000 J/m2) and the expression of hyp53 in the skin, spleen, and liver was analyzed by qPCR. (PPTX 37 kb) [file 12863_2020_856_MOESM4_ESM.pptx]
