# Supplementary material for: Cloning of Hynobius lichenatus (Tohoku hynobiid salamander) p53 and analysis of its expression in response to radiation
Source: BMC Genet. 2020 May 20;21:53. doi: 10.1186/s12863-020-00856-0 (PMC7238597; doi:10.1186/s12863-020-00856-0)
Supplement: Supplementary file 5 — Additional file 5. The original images of western blot analysis. (A) The original image of western blot analysis showed in Fig. 5. (B) The original image of western blot analysis showed in Fig. 6a [file 12863_2020_856_MOESM5_ESM.pptx]

## Slide 1
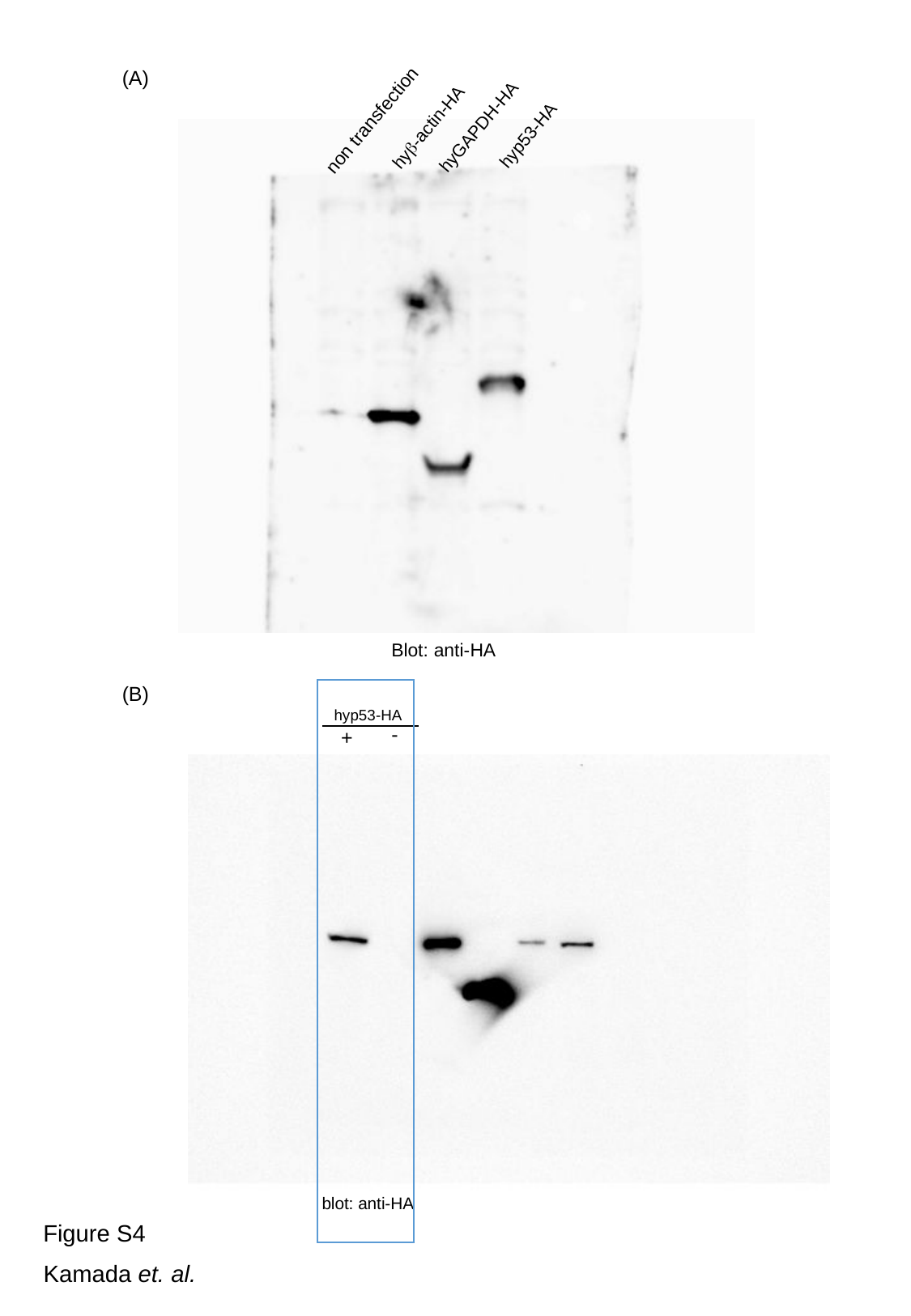

non transfection
hyb-actin-HA
hyGAPDH-HA
hyp53-HA
Blot: anti-HA
(A)
(B)
hyp53-HA
-
+
blot: anti-HA
Figure S4
Kamada et. al.
